# Differential Subcellular Localization of the Splice Variants of the Zinc Transporter ZnT5 Is Dictated by the Different C-Terminal Regions

**DOI:** 10.1371/journal.pone.0023878

**Published:** 2011-08-24

**Authors:** Jared K. Thornton, Kathryn M. Taylor, Dianne Ford, Ruth A. Valentine

**Affiliations:** 1 The Human Nutrition Research Centre, Institute for Cell and Molecular Biosciences, Newcastle University, Newcastle upon Tyne, United Kingdom; 2 School of Dental Sciences, Institute for Cell and Molecular Biosciences, Newcastle University, Newcastle upon Tyne, United Kingdom; 3 Institute for Cell and Molecular Biosciences, Newcastle University, Newcastle upon Tyne, United Kingdom; 4 Tenovus Cancer Research Centre, Welsh School of Pharmacy, Cardiff University, Cardiff , United Kingdom; University of Cambridge, United Kingdom

## Abstract

**Background:**

Zinc is emerging as an important intracellular signaling molecule, as well as fulfilling essential structural and catalytic functions through incorporation in a myriad of zinc metalloproteins so it is important to elucidate the molecular mechanisms of zinc homeostasis, including the subcellular localizations of zinc transporters.

**Principal Findings:**

Two splice variants of the human *SLC30A5* Zn transporter gene (ZnT5) have been reported in the literature. These variants differ at their N- and C-terminal regions, corresponding with the use of different 5′ and 3′ exons. We demonstrate that full length human ZnT5 variant B is a genuine transcript in human intestinal cells and confirm expression of both variant A and variant B in a range of untreated human tissues by splice variant-specific RT-PCR. Using N- or C-terminal GFP or FLAG fusions of both isoforms of ZnT5 we identify that the differential subcellular localization to the Golgi apparatus and ER respectively is a function of their alternative C-terminal sequences. These different C-terminal regions result from the incorporation into the mature transcript of either the whole of exon 14 (variant B) or only the 5′ region of exon 14 plus exons 15–17 (variant A).

**Conclusions:**

We thus propose that exons 15 to 17 include a signal that results in trafficking of ZnT5 to the Golgi apparatus and that the 3′ end of exon 14 includes a signal that leads to retention in the ER.

## Introduction

Zinc is an essential micronutrient with widespread roles in human health, resulting from the prevalence of zinc-containing proteins (comprising 3–10% of the human genome) [1] with diverse functions. Recent studies provide evidence that extracellular stimuli can affect intracellular free zinc concentrations, including through a rapid release of stored intracellular zinc – the “zinc wave” – with effects on cell function [2], [3], [4]. Zinc is thus emerging as novel intracellular second messenger. It is therefore important to elucidate the molecular mechanisms of mammalian zinc homeostasis.

Zinc cannot pass through biological membranes by simple passive diffusion and therefore zinc transport proteins are essential to mediate cellular zinc uptake and efflux as well as intracellular zinc sequestration to maintain cellular zinc homesostasis. Membrane transport proteins with zinc transport capacity comprise two major, families classified as SLC30 (ZnT family) and SLC39 (ZIP family). In general, ZnT family proteins mediate cellular zinc efflux or intracellular sequestration within membrane-bound compartments/organelles while ZIP family proteins operate in the opposite direction [5]. However, there are clearly examples of proteins in both families that can operate either bi-directionally or counter to the usual direction for other family members [6], [7], [8]. To date 10 ZnT proteins and 14 ZIP proteins have been identified in humans, and the expression and localization of each varies depending upon the cell type.

**Figure 1:**
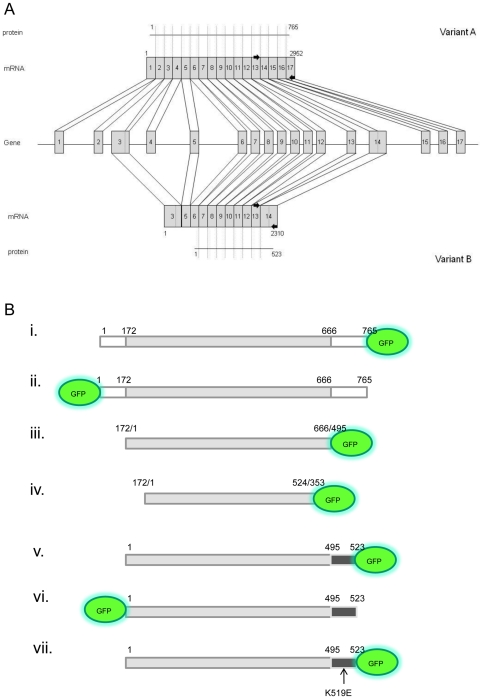
Schematic diagram depicting the *ZnT5* gene and the combinations of exons comprising variants A and B and illustrating the fusions and truncated protein constructs of ZnT5 generated. (A) The *ZnT5* gene consists of 17 exons, all (with the exception of the 5′end of exon 3 and the 3′ end of exon 14) are included in the mRNA for variant A. Exclusion of exons 1, 2, 4 and 15–17, plus inclusion of the 5′end of exon 3 and the 3′ end of exon 14, gives rise to variant B mRNA. A single primer that leads to PCR amplification of both variants anneals to sequence within exon 13. Reverse primers anneal to the unique 3′ ends of variant A and variant B. Black arrows indicate primers. (B). Diagram illustrating fusion and truncated protein constructs of ZnT5. The grey box represents the region of identity between variants A and B. The white boxes represent regions specific to variant A. Black boxes represent regions specific to variant B. Location of the GFP tag at the N- or C- terminus is shown. Numbering corresponds to amino acids in either variant A or variant B and shows points of divergence between the two isoforms. All constructs were also made with FLAG epitope tags.

Two splice variants of the human *SLC30A5* Zn transporter gene (ZnT5) have been reported in the literature [9], [10]. The sequences reported differ at their N- and C-terminal regions, corresponding with the use of different 5′ and 3′ exons [11] and (Figure 1A). The ZnT5 splice variants adopted different subcellular localizations when expressed as fusions to GFP from the corresponding transgenes introduced into Chinese hamster ovary cells. Variant A was expressed in the Golgi apparatus whereas variant B was expressed throughout the cell, including at the plasma membrane [11]. Plasma membrane localization of variant B, specifically localization at the apical membrane, has also been observed in human intestinal Caco-2 cells [9], and we have also reported previously localization of ZnT5 to the apical enterocyte membrane in human small intestine, using an antibody that may potentially recognize both splice variants [12]. There has been a suggestion that expression levels of ZnT5 variant B are low or that the transcript can be detected only after stimulation in specific cell types, based on an observation that, under certain conditions, variant B cDNA was difficult to generate by RT-PCR and on observations that variant B is not found in any mRNA/cDNA databases [13]. Here, full length ZnT5 variant B is confirmed as a genuine transcript in human intestinal Caco-2 cells grown under standard conditions for 24 hours, using simple RT-PCR based techniques. Furthermore, using primers specific to the unique 3′ sequences in both variant A and variant B, expression of both isoforms is confirmed in a range of untreated human tissues by RT-PCR.

The targeting sequences and mechanisms responsible for the differential subcellular localization of the two ZnT5 variants have yet to be defined. Recent evidence suggests that the (predicted) cytosolic C-terminal tail of ZnT5 variant A is important for heterodimerisation with the zinc transporter ZnT6. These studies indicated that the N-terminal region of ZnT5 is not involved in the interaction with ZnT6, based on co-localization with ZnT6 to a vesicular compartment of ZnT5 expressed with an N-terminal deletion [13]. To investigate the potential role of the different N- and C- terminal domains of the ZnT5 splice variants in differential subcellular localisaton, we created a series of tagged ZnT5 constructs; these constructs varied in the position of the tag (N-terminal, C-terminal) and the tag identity (GFP or FLAG epitope) and in the presence or absence of the splice variant-specific N- and C-terminal regions of the protein (Figure 1B). We examined the localization of each expressed fragment in Hela cells, HEK293 cells and Caco-2 cells. Subcellular localizations were consistent in all cell models and were not affected by the nature of the fused protein tag. Images revealing patterns of subcelluar localization herein are shown for GFP constructs only, expressed in Hela cells.

## Results

**Figure 2:**
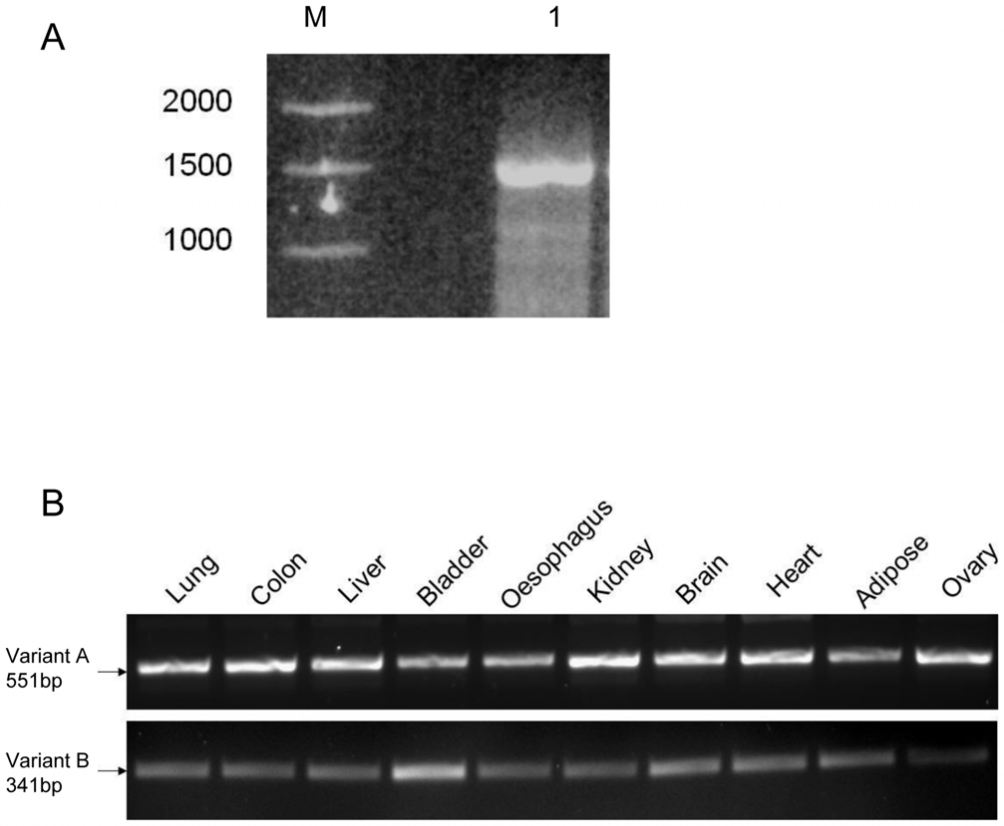
Expression of splice variants of ZnT5 detected by RT-PCR. (A) Variant B is a genuine transcript of the *ZnT5* gene. Product corresponding to a full-length ZnT5 variant B mRNA (1569 bp, lane 1) was generated by RT-PCR from total RNA extracted from human intestinal Caco-2 cells grown for 24 hours under standard conditions, which does not promote polarisation. Identity to the expected product was confirmed by sequencing. Molecular weight markers with sizes indicated shown (lane M). (B) Tissue-specific RT-PCR carried out using primers designed to anneal to the unique 3′ end of variant A (exons 13–17) and variant B (exons 13–14 ^3′^) in a multiple tissue RNA panel (Ambion) revealed ubiquitous expression of both transcripts. Identity to the expected products was confirmed by sequencing. Negative control RT-PCR reactions identical to those yielding the products shown except for the omission of Moloney murine leukemia virus reverse transcriptase generated no products. Control reactions for all samples using primers to GAPDH [11] were including to control for input (results not shown).

Given that questions have been raised about the existence of a splice variant of ZnT5, designated ZnT5 variant B [13], we sought to explore further the representation of the corresponding mRNA species in total RNA extracted from human intestinal Caco-2 cells and from a range of human tissues. Our previous data revealing the ubiquitous existence of ZnT5 splice variant B in a variety of human tissues relied on northern blotting and use of either a variant A-specific or non-variant-specific probe [11] (A probe that hybridises only to the unique sequence regions of variant B could not be generated). We thus generated PCR primers specific to the individual ZnT5 splice variants to confirm the existence of both. Use in RT-PCR of primers designed to amplify specifically full-length ZnT5 variant B mRNA resulted in a product of the expected size (1569 bp) from total RNA extracted from human intestinal Caco-2 cells grown for 24 hours under standard conditions, which does not promote polarisation (Figure 2A). Sequencing revealed identity to the expected product. The nucleotide sequence has been submitted to the GenBank™/EBI Data Bank with accession number JF802034. These data confirm the existence and expression of ZnT5 variant B in Caco-2 cells under the cell culture conditions employed.

RT-PCR carried out using primers designed to anneal to the unique 3′ end of variant A (exons 13–17) and variant B (exons 13–14^3′^) carried out on a multiple tissue RNA panel (Ambion) revealed ubiquitous expression of both transcripts, whose identity to the expected products was confirmed by sequencing. The varying intensities of bands in both panels indicates that the PCR had not reached saturation and therefore we have been able to compare different tissues with respect to their relative ratio of variant B to variant A. (Comparison of absolute ratios cannot be achieved because the different primer pairs used to generate products may have resulted in different amplification efficiencies). Levels of the transcripts differed between different tissues, as already reported [10], [11], (Figure 2B). Compared with other tissues, we observed that RNA from bladder contained a notably higher relative ratio of ZnT5 variant B to variant A, with a similar, though less marked, relationship also observed for brain, heart and adipose tissue. Along with our previous analyses [11], these data demonstrate robustly that ZnT5 splice variant B is a genuine transcript of the *SLC30A5* gene.

Previous reports of the subcellular localization of ZnT5 based on the use of antibodies with the potential to cross-react with both variants [10], [12] have identified subcellular localization to either the Golgi apparatus in HEK293 cells [10] or apical enterocyte membrane in the intestine [12]. Further, evidence based on transient expression of either variant A or variant B conjugated to marker proteins at the C-terminus revealed, for variant A, localization to the Golgi apparatus [11] or the plasma membrane in unpermeabilized Caco-2 cells [8] and, for variant B, diffuse localization, including co-localization with wheat germ agglutinin staining, in unpermeabilized CHO cells, indicating some localization at the plasma membrane [11]. To elucidate more fully the localization of the splice variants of ZnT5, we generated constructs including both N- and C- terminal GFP and FLAG epitope tags and visualized the expression of the recombinant protein in three different cell types: HeLa, HEK293 and Caco-2. The pattern of localization did not vary with cell type or with identity of the tag, thus for clarity we show here only data for HeLa cells expressing GFP-tagged proteins. Data based on the use of FLAG-tagged constructs are shown in the Figure S1.

**Figure 3:**
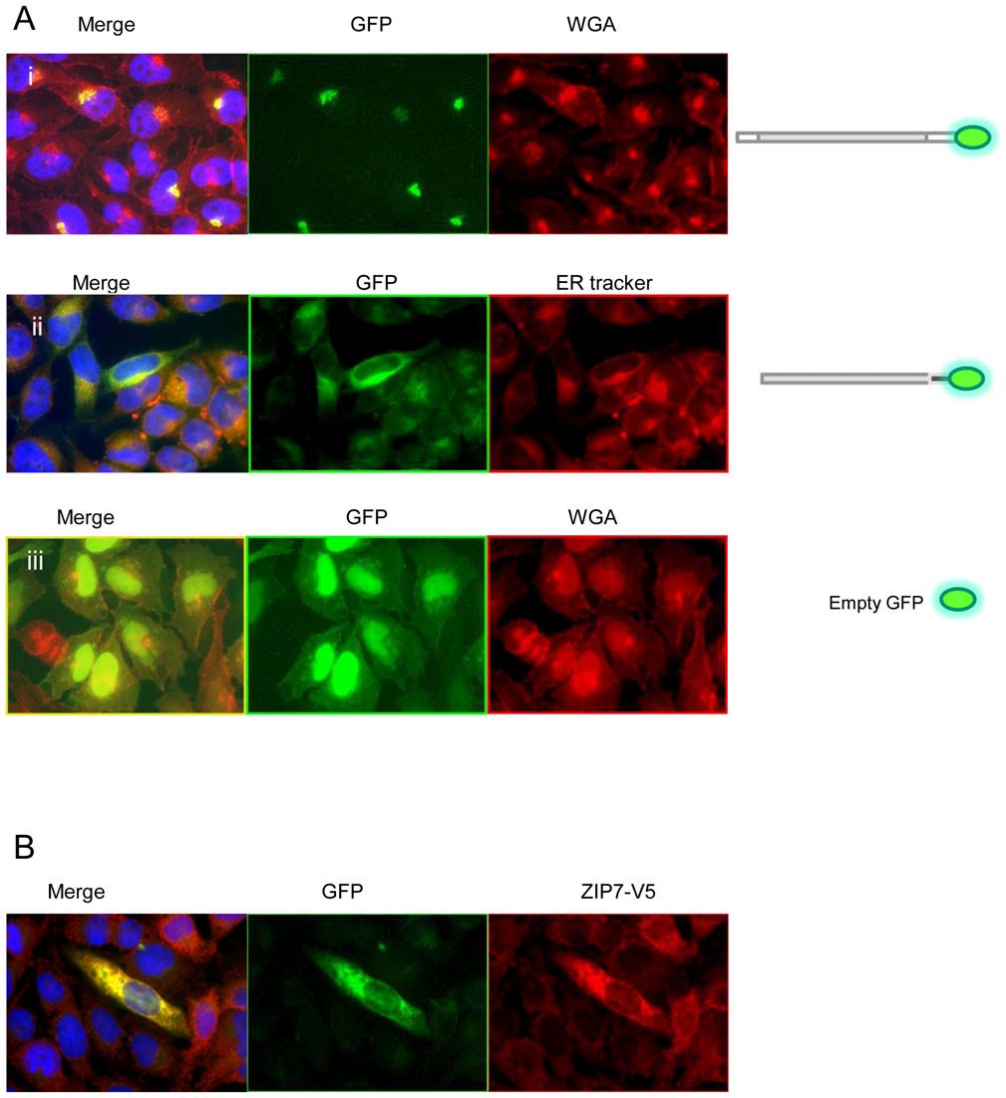
Subcellular localization of variants of ZnT5 expressed with a C-terminal GFP tag in HeLa cells. (**A**) (i)Variant A localizes to the Golgi apparatus, as revealed by co-localization with Rh-labelled wheat germ agglutinin (WGA) in permeabilized cells. (ii) Variant B localizes to the ER, as revealed by co-localization with Rh-labelled ER Tracker. (iii) GFP expressed without a protein fusion (negative control). Diagram illustrating C-terminal fusion constructs of ZnT5 shown alongside. (**B**) Co transfection of ZnT5 variant B expressed with an N-terminal GFP tag (green) and ZIP7 with a C-terminal V5 tag (red) showing co-localization to the ER. In merged images shown in A (i) and (ii) nuclei are stained with DAPI (blue).

Expression of ZnT5 variant A with a C- terminal GFP tag (Figure 1B i) in permeabilized HeLa cells confirmed localization at the Golgi apparatus, as reported previously [10], [11]; Figure 3A i. This construct did not co-localize with Rh-labelled ER Tracker (results not shown), so does not localize to the ER. GFP fused to the C-terminal region of ZnT5 splice variant B (Figure 1B v) showed co-localization with a Rh-labelled ER Tracker in permeabilized Hela cells, consistent with localization to the endoplasmic reticulum (ER) (Figure 3A ii). This construct did not co-localize with Rh-labelled WGA (results not shown), so does not localize to the Golgi apparatus. GFP–only control showed a diffuse pattern of expression throughout the cell in all experiments (Figure 3A iii), confirming that localization of the signal to specific subcellular regions was a function of the attached ZnT5 protein or protein fragment. Further assurance that observed patterns of localization did not reflect any property or function of the GFP tag was provided by the observation that, as already noted, identical patterns of localization were obtained using equivalent constructs but with proteins tagged with the FLAG epitope, detected using anti-FLAG antibody. For all constructs, there was no alteration in the pattern of localization in response to changing the extracellular zinc concentration for 24 h immediately prior to viewing the cells either by adding ZnCl_2_ to 150 µM or adding the membrane-permeable zinc chelator TPEN (5 µM) (data not shown); thus, we find no evidence for zinc-induced intracellular trafficking of either variant of ZnT5, consistent with previous observations [11].

To seek further confirmation that ZnT5 variant B is expressed at the membrane of the ER, we made use of the fact that localization of the zinc transporter ZIP7 to the ER is well-established [14]. Cells were co-transfected with the construct driving expression of ZnT5 variant B conjugated at the N-terminus to GFP with a construct driving expression of ZIP7 with a C-terminal V5 epitope tag, which was detected using anti-V5 monoclonal antibody followed by an anti-mouse IgG secondary polyclonal antibody conjugated to AlexaFluor 594. The GFP and AlexaFluor 594 signals showed clear co-localization (Figure 3B), demonstrating that both zinc transporters are expressed at the same subcellular membrane.

**Figure 4:**
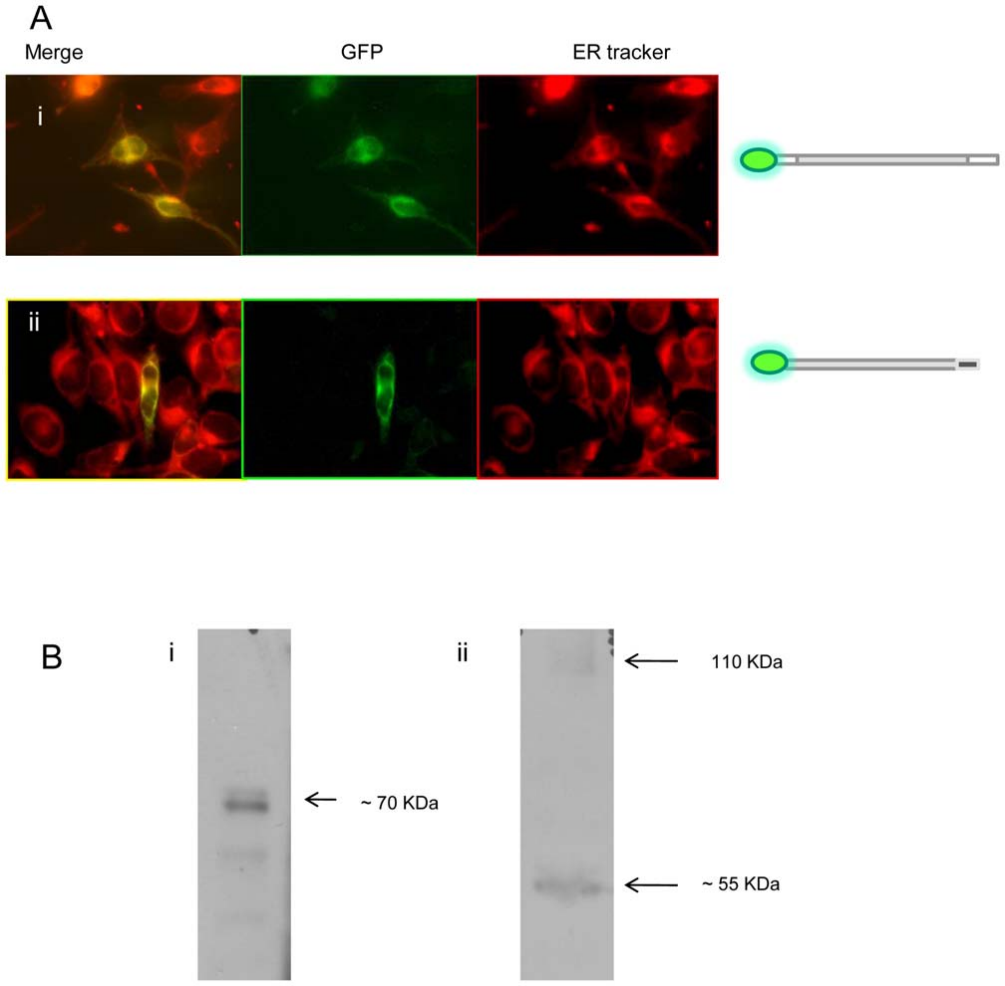
Subcellular localization of variants of ZnT5 expressed with an N-terminal GFP tag in HeLa cells and expression in HeLa cells of ZnT5 proteins fused with GFP at either the C- or N- terminus visualized by immunoblotting. (A) (i) Localization of variant A expressed with GFP fused to the N-terminus was observed at the ER, confirmed by co-localization with Rh-labelled ER Tracker. (ii) Localization of variant B expressed with GFP fused to the N-terminus was observed at the ER, confirmed by co-localization with Rh-labelled ER Tracker. Diagram illustrating N-terminal fusion constructs of ZnT5 shown alongside. (B) (i) Immunoblotting using an antipeptide antibody against GFP in cells expressing variant A with GFP fused to the C-terminus. (ii) Immunoblotting using antipeptide antibody against GFP in cells expressing variant A with GFP fused to the N-terminus. Approximate band sizes given using comparison to a prestained broad range protein marker (NEB).

Intriguingly, and in contrast to the pattern of expression we observed with GFP fused to the C-terminus (Golgi apparatus), localization of variant A expressed with GFP fused to the N-terminus (Figure 1B ii) was observed at the ER, confirmed by co-localization with ER Tracker (Figure 4A i). The presence of an N-terminal cleavage site could explain this pattern of localization based on a model in which a mature, cleaved peptide trafficks to the Golgi apparatus, while the cleaved N-terminal fragment is retained in the ER. Consistent with this theory, Western blot analysis using an anti-GFP antibody revealed a protein band of approximately 70–80 KDa in total protein extract from HeLa cells transfected with the construct from which ZnT5 variant A was expressed fused at the C-terminus to GFP, consistent with a cleaved product size (expected size of the un-cleaved fusion protein (including GFP) 111 KDa). In further support of this model, a protein product of approximately 55 KDa was revealed by anti-GFP antibody on a Western blot of total protein extract from HeLa cells transfected with the construct from which ZnT5 variant A was expressed fused at the N-terminus to GFP. A further faint band of 111 KDa, consistent with the presence of some residual, uncleaved protein, was also observed (Figure 4B).

In contrast to the differential patterns of localization observed for ZnT5 variant A, depending on the position of the GFP or FLAG tag, ZnT5 variant B localization to the ER was independent of whether the GFP or FLAG tag was on the N- or C-terminus (Figure 1Bvi; Figure 4A ii), consistent with a model in which either the variant B peptide does no undergo any cleavage or where both sections of a cleaved product localize to the ER.

**Figure 5:**
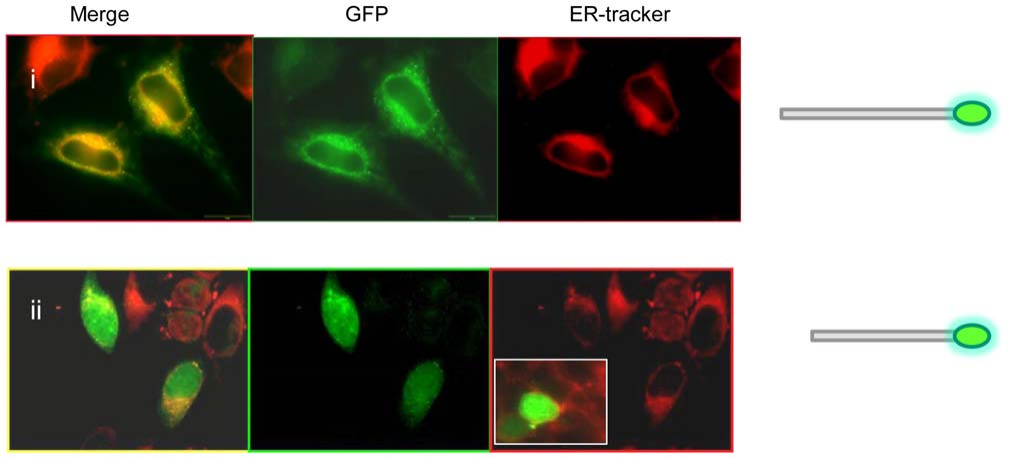
Correct localization of ZnT5 requires the region 525–666 according to amino acid numbering of Variant A, containing the histidine-rich segment, plus the C-terminal region specific to variant A or B. (A) Hela cells expressing transiently a construct of ZnT5 representing the region ‘core’ to both variants (amino acids172–666) with a C-terminal GFP tag. The ER is stained (red) with ER Tracker. Note that localization overlaps with the ER stain, but also includes punctuate structures. Diagram illustrating truncated protein construct 172–666 of ZnT5 shown alongside (B) Hela cells expressing transiently a construct of ZnT5 comprising the region 172 to 524 with a C-terminal GFP tag. In (ii) the ER is stained (red) with ER Tracker. The inset panel shows the Golgi apparatus stained (red) using WGA. Note that the pattern of localization overlaps with but is not restricted to the Golgi apparatus or ER. Diagram illustrating truncated protein construct 172–524 of ZnT5 shown alongside.

**Figure 6:**
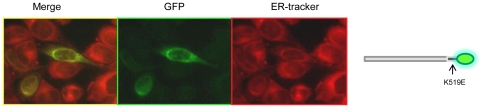
A mutated construct containing the full length version of variant B, with a substitution mutation at position 519 of a potential ER retention motif (K519E), does not disrupt the ER localization. Diagram illustrating C-terminal fusion construct of ZnT5 shown alongside.

Based on the observation that the two alternative transcripts of ZnT5 incorporate different C-terminal regions, corresponding with the use of different 3′exons [11], we hypothesized that differential localization may be dependent on this region. To test this hypothesis we generated two constructs of ZnT5, one for expression of only the region of the protein that is common to both variants A and B (amino acids 172–666, according to residue numbering for variant A) with a C-terminal GFP tag (Figure 1B iii) and one lacking the histidine rich region common to both variants and also lacking Asp599, which has been shown to be involved in zinc binding in variant A [15] (amino acids 172–524, according to residue numbering for variant A) with a C-terminal GFP tag (Figure 1B iv). Use of the ‘core’ ZnT5 construct (amino acids 172–666, Figure 1B iii) revealed localization to the ER, however a punctuate/vesicular staining pattern was also observed, not seen with either full length version of ZnT5 (Figure 5i). Use of the shorter construct lacking the histidine rich region and Asp599 (amino acids 172–524, Figure 1B vi) revealed no localization of ZnT5 to the Golgi apparatus or the ER (no co-localization with WGA or ER Tracker dye) and a diffuse pattern of localization similar to that observed on transfection with a plasmid from which GFP was expressed without any protein fusion (negative control) (Figure 5ii). The observations are, thus, consistent with a model in which the region including amino acids 525 to 666, encompassing the histidine-rich region, is required for retention within the system of membranes that comprise the ER and Golgi apparatus, and in which the variant A-specific C-terminal region, but not the variant B-specific C-terminal region, directs trafficking of the protein to the Golgi apparatus (probably following cleavage to remove a signal peptide at the N-terminal region). This model was further confirmed using an N-terminal deletion (amino acids 1–171) construct of variant A (comprising amino acids 172–765) with a C-terminal GFP tag. This construct had an identical pattern of expression to full length variant A tagged at the C-terminus with GFP (i.e. Golgi localization), (result not shown). Of possible relevance to retention of ZnT5 variant B in the ER, a motif very close to a consensus ER retention motif (consensus K (K/Q) XX, when comprising the 4 C-terminal-most amino acid residues) specific to variant B (KQTTK) at the 5 C-terminal-most amino acid residues (519–523) was identified using the Signal P algorithm. A mutated construct containing the full length version of variant B, with a substitution mutation at position 519 (i.e – 4 from the carboxy terminus) of this ER retention motif (K519E) (Figure 1 B v) did not disrupt localization to the ER (Figure 6). These observations indicate that the region identified is not involved in retention of ZnT5 variant B in the ER and that other differences between the variant A and B carboxy-terminal regions dictate trafficking to the Golgi apparatus or retention in the ER, respectively. Work to identify specific residues present in the C-terminal regions responsible for localization of variant A to the Golgi apparatus and variant B to the ER is ongoing.

## Discussion

Alternative splicing of the ZnT5 gene gives rise to two isoforms with unique N- and C-termini. It has been noted that variant B cDNA was not easily generated by RT-PCR from Caco-2 cell RNA, suggesting a possible low expression level that may only increase in response to stimulus in specific cell types [13]. Here, full length ZnT5 variant B is confirmed as a genuine transcript in unpolarised Caco-2 cells using simple RT-PCR based techniques. Furthermore, using primers specific to the unique 3′ sequences in both variant A and variant B, expression of both isoforms was confirmed in a range of human tissues by RT-PCR. Although the data do not allow comparison of absolute ratios, with respect to differences between tissues, greatest expression of variant B relative to variant A was detected in bladder, and brain, heart and adipose tissue also appeared to express variant B at relatively high levels. The ubiquitous expression of both isoforms would be consistent with a wide-ranging role for both variant A and variant B in zinc homeostasis.

**Figure 7:**
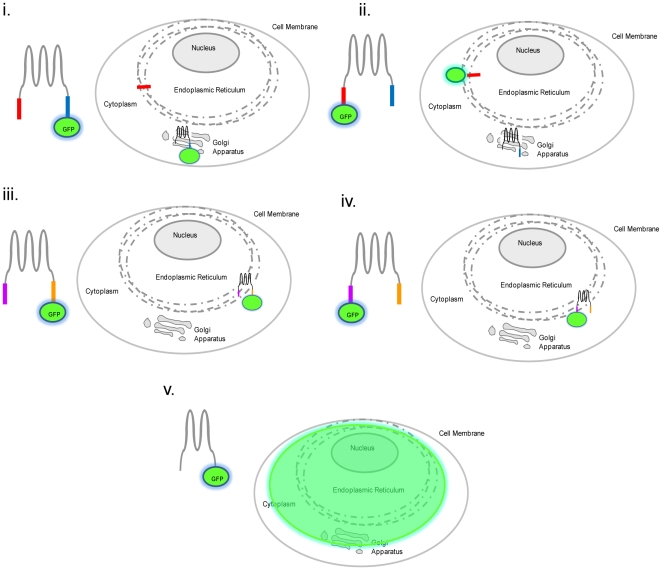
Schematic diagram of a model for localization of ZnT5. Variant A is expressed as a pro-protein that undergoes cleavage in the ER to remove an N-terminal signal peptide, which is retained in the ER, while the mature, processed protein trafficks to the Golgi apparatus. In contrast, variant B is retained in the ER. Thus, when variant A is detected by virtue of a GFP (or epitope) tag attached to the C-terminus, the signal is observed at the Golgi apparatus (i). If variant A is detected by virtue of a GFP (or epitope) tag attached to the N-terminus, however, the signal is observed at the ER (ii). In the case of variant B, which does not undergo any proteolytic cleavage, the signal is observed at the ER irrespective of whether the GFP (or epitope) tag is at the C-terminus (iii) or N-terminus (iv). Retention within the ER/Golgi apparatus requires the region 525 to 666, so a diffuse signal is observed when the protein is detected by virtue of a GFP (or epitope) tag attached (at either the N- or C-terminus) to a truncated protein lacking this region (v). Note that there is no intention that the toplogical representation of the protein is accurate.

Localization of ZnT5 variant A expressed with a C- terminal GFP or FLAG tag was confirmed at the Golgi apparatus, consistent with previous reports [10], [11], however the same variant expressed as an N-terminal GFP or FLAG fusion was observed at the ER, co-localizing with ER Tracker. We propose the presence of an N-terminal cleavage signal and suggest that the form of ZnT5 variant A observed in the Golgi apparatus is a mature cleavage product of a pro-peptide and that the cleaved N-terminal region is retained in the ER (and so observed here when the GFP or FLAG tag is at the N-terminus). This model is presented schematically as Figure 7. Western blot analysis further supports this theory, with a band of approximately 70–80 KDa being detected by anti-GFP antibody in protein prepared from cells expressing the C-terminally tagged construct, whereas the N-terminally tagged construct was visualized at approximately 40–55 KDa (consistent with the size of the cleaved N-terminal fragment plus GFP). A faint band of 110 KDa is visible on this blot, which could possibly be explained as unprocessed native protein (Figure 4B). Consistent with this model, it was reported previously that endogenous ZnT5 variant A protein detected by immunoblot analysis was smaller than the expected calculated size, and it was suggested that ZnT5 variant A may be cleaved to yield a mature protein [10]. Furthermore, recent studies have identified that ZnT5 variant A expressed heterologously with FLAG fused to the N-terminus appeared smaller (55 kDa) than the predicted size (79 kDa) on immunoblot analysis using an anti-hZnT5 antibody [15]. Our observation agrees with recent evidence provided by Fukunaka and colleagues who found that a FLAG-tagged N-terminal deletion mutant of ZnT5 variant A that was complete at the C-terminus co-localized with HA-hZnT6 to vesicular compartments, indicating that the N-terminal region of ZnT5 is not required for its subcellular localization [13]. These results, taken with our current data showing lack of localization of ZnT5 with a C-terminal deletion, indicate that the C-terminal region must be involved in targeting the mature peptide to the Golgi apparatus. A substantial body of evidence is taken to demonstrate that ZnT5 variant A plays a role in the Golgi apparatus to deliver zinc to nascent zinc-containing proteins, and can act in this capacity as a functional heterodimer with ZnT6. Such evidence has been derived from approaches including expression of the recombinant protein. Such data should be interpreted considering the likelihood that the functional ZnT5 variant A protein in the Golgi apparatus results from cleavage of a pro-peptide [13], [15], [16].

The novel observation that ZnT5 variant B localizes to the ER raises intriguing questions about a potential role of variant B in ER-mediated zinc homeostasis. As already noted, ZIP7 has been localized to the ER membrane and has been predicted to have a specialised function in transporting zinc from the ER into the cytosol [14], providing the basis of intracellular zinc signalling when released as the “zinc wave” [2], [3], [4], [17]. Several pieces of recent data are suggestive of a mechanism for zinc distribution in cells, whereby extracellular zinc entering the cell is immediately buffered within a‘zinc muffler’ and translocated into an intracellular zinc store, such as the ER [18], [19] before ZIP7 releases zinc into the cytosol from the ER [20] in the form of the ‘zinc wave’ [2], [3], [4]. The identity of the zinc transporter responsible for the translocation of zinc into the ER from the ‘zinc muffler’ is still unknown. Our novel observation that ZnT5 variant B co-localizes with ZIP7 to the ER, in view of our previous findings that this transporter can operate both in uptake and efflux mode [8], suggests that it may fulfill this role [14], [17]. Two categories of ER retention/retrieval motifs have been characterised. The C-terminal tetrapeptide KDEL sequence has been shown to be necessary for retention of soluble ER-resident proteins (reviewed by [21]), but is not found in ZnT5. A sequence almost matching the consensus for a second ER-retention motif (K (K/Q) XX, when comprising the 4 C-terminal-most amino acid residues) is present at the C-terminus of ZnT5 variant B (KQTTK, so deviating from the consensus motif by having an additional C-terminal residue). Mutation of the lysine reside to alanine did not affect localization, so the motif appears to be unrelated to retention in the ER in this case. It is not without precedence that ER localized proteins include no identifiable or consensus ER retention motif; for example, subunits of the Sec61 protein are detected to localize to the ER, despite the fact that none of them contain any known membrane protein retrieval signal such as cytosolic di-lysine or N-terminal di-arginine motifs [22]. Furthermore, ZIP7 does not contain an obvious ER retention motif, apart from ARGL in the N-terminal signal peptide, which would presumably be cleaved after processing in the ER [14].

We reported previously localization of ZnT5 to the human enterocyte apical membrane and to the equivalent location in polarized human intestinal Caco-2 cells, using an antibody likely to be cross-reactive with both splice variants [12]. Similarly, we observed apical membrane localization of heterologously-expressed, epitope-tagged ZnT5 variant B in polarized Caco-2 cells [9]. The fact that we observed no plasma membrane localization of ZnT in HEK293 or HeLa cells in the current study may be related to a specific localization mechanism that accompanies cell polarization. We also reported plasma membrane (as well as diffuse intracellular) localization of ZnT5 variant B based on heterologous expression of a GFP-tagged protein in CHO cells [11], pointing towards cell-type or species-specific mechanisms of localization.

In summary, we present further robust evidence for the existence of two major splice variants of the *SLC30A5* gene, resulting in ZnT5 variants A and B, and we identify that differential subcellular localization to the Golgi apparatus and ER respectively is a function of their alternative C-terminal sequences. These different C-terminal regions result from the incorporation into the mature transcript of either the whole of exon 14 (variant B) or only the 5′ region of exon 14 plus exons 15–17 (variant A) (Figure 1A). We thus propose that exons 15 to 17 include a signal that results in trafficking of ZnT5 to the Golgi apparatus and that the 3′ end of exon 14 includes a signal that leads to retention in the ER.

## Materials and Methods

### Plasmid construction

Full-length cDNA corresponding to ZnT5 splice variant B was generated by RT-PCR from total RNA extracted from Caco-2 cells by RT-PCR using Velocity DNA polymerase (Bioline) with primers and thermal cycling parameters as stated in Table 1. For variant A the plasmid pEGFP-ZnT5A [11] was used (re-named pZnT5A-GFP (Figure 1B i)), or used as a template for the GFP fusion at the N-terminus. For expression of full-length ZnT5 variant B with a GFP fusion at the C-terminus the PCR product was subcloned in-frame into the vector pEGFPN (*Sac*I and *Apa*I) (Clontech), to give the plasmid pZnT5B-GFP (Figure 1B v). For expression of ZnT5 splice variants with a GFP fusion at the N-terminus PCR products were subcloned in-frame into the vector pEGFPC (*Eco*RI and *Apa*I) (Clontech), to give the plasmids p-GFPZnT5A (Figure 1B ii) and pGFPZnT5B (Figure 1B vi). Full length ZnT5 was also subcloned into the FLAG vector (Sigma) to give the plasmid pFLAG-ZnT5, for expression with an N-terminal FLAG epitope (*Not*I and *Eco*RV), and pZnT5-FLAG, for expression with a C-terminal FLAG epitope (*Not*I and *Eco*RV). A construct for expression of only the region shared by both splice variants of ZnT5 (Figure 1B iii) or a shorter shared region minus the common histidine rich region (Figure 1B iv) with a C-terminal GFP tag was made using the pZnT5-GFP construct as a template to generate the required PCR product, using High Fidelity ‘Velocity’ DNA polymersase (Bioline) and primers and thermal cycling conditions as listed in Table 1, and was then subcloned in frame into the pEGFPN vector. Correct insert orientation and sequence fidelity was confirmed by sequencing (MWG Biotech). The lysine at position 519 of pZnT5B-GFP was mutated to a glutamate (Figure 1B vii) using the QuikChange Site-Directed Mutagenesis kit (Stratagene) using primers as listed in Table 1. The mutated construct was sequenced to confirm successful mutation (MWG Biotech).

**Table 1:** Primers and cycling parameters for PCR.

Product	Primer sequence	Product Size (bp)	Cycling Parameters
ap-GFPZnT5A	^201^ GATGGAGGAGAAATACGGCG ^220^ ^2496^ ATGATGTAAGTACCATCTTTGC ^2474^	2295	96°C – 30 s, 56°C – 30 s, 72°C – 90 s, 35 cycles
a Variant A cDNA – 3′ end	^1942^ GCGGGTGGAGGCATGAATGCTA ^1963^ ^2496^ ATGATGTAAGTACCATCTTTGC ^2474^	551	95°C – 30 s, 60°C – 30 s, 72°C – 60 s, 25 cycles
a Variant A (172–666)-GFP clone	^714^ CATGGCTAAAATGGCTGAACAC ^735^ ^2199^ CTTTTCTAAAGCAATATGTAGTTCT ^2175^	1485	96°C – 30 s, 56°C – 30 s, 72°C – 90 s, 35 cycles
a Variant A (172–524)-GFP clone	^714^ CATGGCTAAAATGGCTGAACAC ^735^ ^1770^ TGTTAACATGTGAGTGTCTAATTC ^1747^	1056	94°C – 30 s, 57°C – 60 s, 72°C – 90 s, 35 cycles
b Full length variant B cDNA/pZnT5B-GFP	^1^ ATGGCTAAAATGGCTGAACAC ^22^ ^1569^ TTTGGTTGTCTGTTTTACTTCCAG ^1546^	1569	96°C – 30 s, 60°C – 30 s, 72°C – 90 s 35 cycles
b P-GFPZnT5B	^1^ ATGGCTAAAATGGCTGAACAC ^22^ ^1569^ TTTGGTTGTCTGTTTTACTTCCAG ^1546^	1569	96°C – 30 s, 60°C – 30 s, 72°C – 90 s 35 cycles
b Variant B cDNA – 3′ end	^1228^ GCGGGTGGAGGCATGAATGCTA ^1249^ ^1569^ TTTGGTTGTCTGTTTTACTTC ^1549^	341	94°C – 30 s, 60°C – 30 s, 72°C – 60 s, 30 cycles
Variant B (K519E)	^1^ ATGGCTAAAATGGCTGAACAC ^22^ ^1569^ TTTGGTTGTCTGTTCTACTTCCA ^1547^	1569	96°C – 30 s, 60°C – 30 s, 72°C – 90 s 30 cycles

Times are given in the order denaturing, annealing, extension and the number of cycles is indicated.

a Numbered according to Genbank™ sequence NM_022902.

b Numbered according to Genbank ™sequence JF802034.

### Semi quantitative RT-PCR

ZnT5 variant A and B transcript abundance, in samples of total RNA prepared from different human tissues (RNA Tissue panel; Ambion), was measured by semi-quantitative RT-PCR using primers specific to ZnT5 variant A and variant B as listed in Table 1. The amplification products from both primer pairs were sequenced (MWG Biotech) to confirm identity to the expected products.

### Subcelluar localization of GFP-tagged and FLAG-tagged constructs

For transfection into Hela cells, all plasmid constructs or GFP/FLAG vector (without insert) were prepared using the EndoFree Plasmid Maxi kit (Qiagen). Hela cells were grown in Dulbecco's modified Eagle's medium containing 10% fetal calf serum, 1% non essential amino acids, (all from Sigma) in 6-well plates on glass coverslips. Cells were transfected 24 h post-seeding, when approximately 50% confluent, using GeneJammer transfection reagent (Stratagene), following the manufacturer's instructions with a ratio of DNA to GeneJammer of 2 µg∶5 µl. Twenty-four hours post-transfection, cells were stained with 1 µM rhodamine labelled ER Tracker dye (Molecular Probes) for 30 minutes according to manufacturer's instructions and then fixed in 4% paraformaldehyde (in phosphate-buffered saline) for 30 min at room temperature, then washed three times with phosphate-buffered saline. If necessary, cells were permeabilized by treatment with 0.5% Triton X-100 for 15 min followed by a further wash with phosphate-buffered saline. Coverslips were mounted in Vectashield mounting medium (Vector Laboratories Ltd) then visualized by fluorescence microscopy using an Olympus BX61 fluorescence microscope. In some experiments cells were co-transfected with pGFP-ZnT5B and ZIP7, expressed as a fusion protein with a 5 KDa V5 tag on the C-terminal end [14]. V5 was visualized using an anti V5 monoclonal antibody (1/250, Abcam ab27671) followed by Alexa Fluor 594-conjugated anti-mouse IgG secondary antibody (1/1000, Molecular Probes) as previously described [14].

### Immunoblotting

Hela cells expressing either the variant A ZnT5 construct fused at the C-terminal to GFP or fused at the N-terminal to GFP were trypsinised and washed in ice cold PBS then lysed in an equal volume of a buffer containing 60 mM Tris-HCl (pH 6.8), 2% sodium dodecyl sulphate and 10% glycerol. Total protein was subjected to SDS-PAGE. Proteins were transferred onto PVDF membrane (GE Healthcare) and blocked with 5% non-fat milk, 0.025 M Tris pH 8, 0.05 M NaCl, 5% normal horse serum (Sigma) before incubation with anti-GFP (1∶1000) antibodies in blocking solution overnight at 4°C. Membranes were washed and incubated for 1 h at room temperature with a horseradish peroxidase – conjugated goat anti-mouse IgG secondary antibody (1∶5000 dilution) (Sigma). Antibody binding was visualized as peroxidise activity using ECL-Plus (GE Healthcare) according to manufacturer's instructions.

## Supporting Information

Figure S1 **Subcellular localization of variants of ZnT5 expressed with either a C-terminal or N-terminal FLAG epitope tag in HeLa cells.** (i)Variant A with a C-terminal FLAG tag (green) localizes to the Golgi apparatus. (ii) Localization of variant A expressed with FLAG fused to the N-terminus was observed at the ER (iii) and (vi) Localization of variant B expressed with either FLAG fused to the C-terminus or N-terminus was observed at the ER, Diagram illustrating fusion constructs of ZnT5 shown alongside. In merged images nuclei are stained with DAPI (blue).
(TIF)
