# Supplementary figures and images for: Differential Subcellular Localization of the Splice Variants of the Zinc Transporter ZnT5 Is Dictated by the Different C-Terminal Regions
Source: PLoS One. 2011 Aug 24;6(8):e23878. doi: 10.1371/journal.pone.0023878 (PMC3161073; doi:10.1371/journal.pone.0023878)

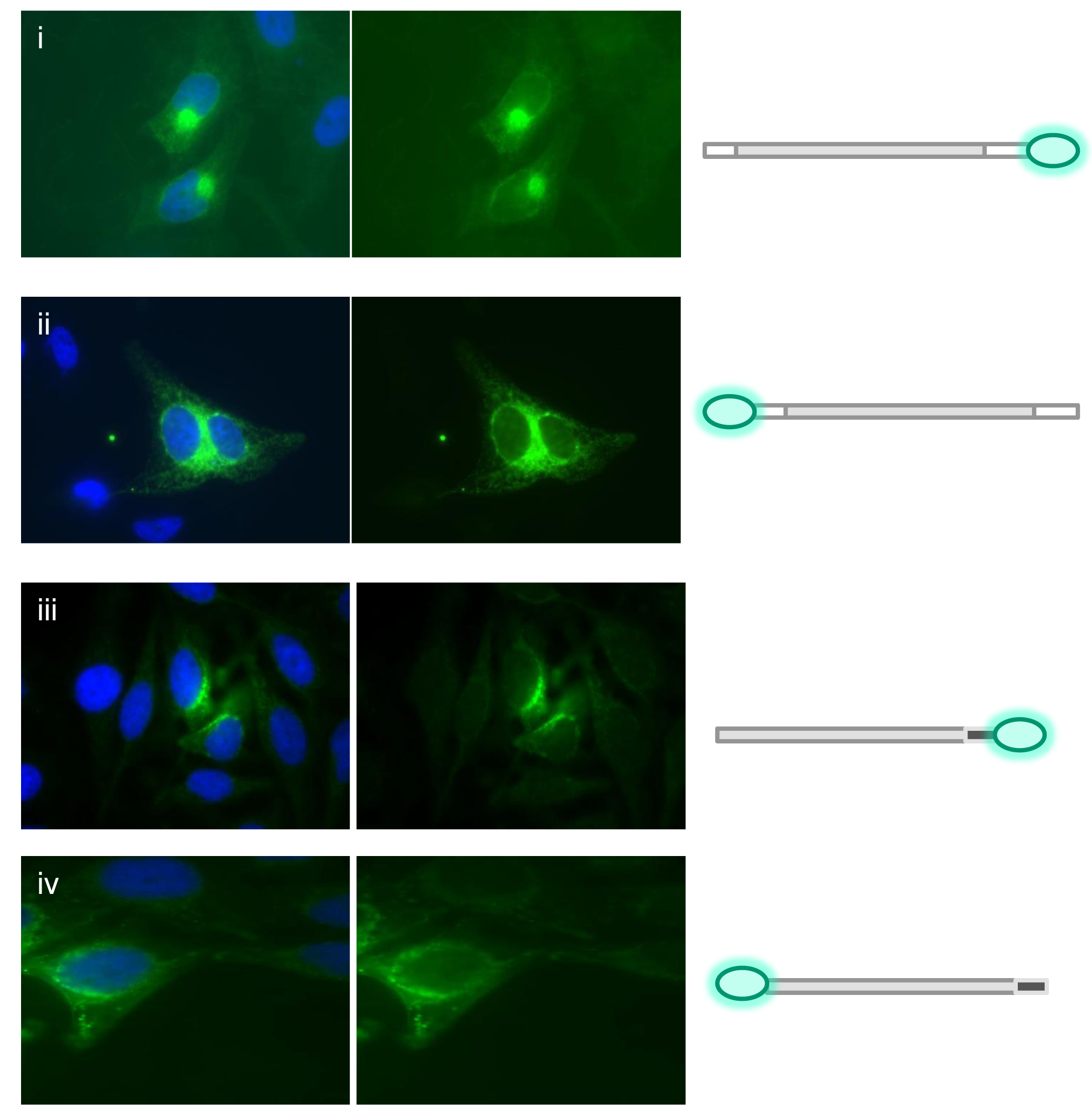

Supplement: Figure S1 — Subcellular localization of variants of ZnT5 expressed with either a C-terminal or N-terminal FLAG epitope tag in HeLa cells. (i)Variant A with a C-terminal FLAG tag (green) localizes to the Golgi apparatus. (ii) Localization of variant A expressed with FLAG fused to the N-terminus was observed at the ER (iii) and (vi) Localization of variant B expressed with either FLAG fused to the C-terminus or N-terminus was observed at the ER, Diagram illustrating fusion constructs of ZnT5 shown alongside. In merged images nuclei are stained with DAPI (blue). (TIF) [file pone.0023878.s001.tif]
